# Characterization of the Distal Promoter of the Human Pyruvate Carboxylase Gene in Pancreatic Beta Cells

**DOI:** 10.1371/journal.pone.0055139

**Published:** 2013-01-30

**Authors:** Ansaya Thonpho, Pinnara Rojvirat, Sarawut Jitrapakdee, Michael J. MacDonald

**Affiliations:** 1 Molecular Metabolism Research Group, Department of Biochemistry, Faculty of Science, Mahidol University, Bangkok, Thailand; 2 Childrens Diabetes Center, University of Wisconsin School of Medicine and Public Health, Madison, Wisconsin, United States of America; Broad Institute of Harvard and MIT, United States of America

## Abstract

Pyruvate carboxylase (PC) is an enzyme that plays a crucial role in many biosynthetic pathways in various tissues including glucose-stimulated insulin secretion. In the present study, we identify promoter usage of the human PC gene in pancreatic beta cells. The data show that in the human, two alternative promoters, proximal and distal, are responsible for the production of multiple mRNA isoforms as in the rat and mouse. RT-PCR analysis performed with cDNA prepared from human liver and islets showed that the distal promoter, but not the proximal promoter, of the human PC gene is active in pancreatic beta cells. A 1108 bp fragment of the human PC distal promoter was cloned and analyzed. It contains no TATA box but possesses two CCAAT boxes, and other putative transcription factor binding sites, similar to those of the distal promoter of rat PC gene. To localize the positive regulatory region in the human PC distal promoter, 5′-truncated and the 25-bp and 15-bp internal deletion mutants of the human PC distal promoter were generated and used in transient transfections in INS-1 832/13 insulinoma and HEK293T (kidney) cell lines. The results indicated that positions −340 to −315 of the human PC distal promoter serve as (an) activator element(s) for cell-specific transcription factor, while the CCAAT box at −71/−67, a binding site for nuclear factor Y (NF-Y), as well as a GC box at −54/−39 of the human PC distal promoter act as activator sequences for basal transcription.

## Introduction

Pyruvate carboxylase (PC) is an important anaplerotic enzyme that catalyzes the ATP-driven carboxylation of pyruvate to oxaloaceate. This reaction is not only the first important committed step of hepatic gluconeogenesis but also crucial for cataplerosis as Krebs cycle intermediates are withdrawn for various biosynthetic purposes including *de novo* fatty acid synthesis in liver and adipose tissue, glyceroneogenesis in adipose tissue and glutamate production in astrocytes (for review see [1]–[3]). PC also plays an important role in normal glucose-stimulated insulin secretion (GSIS) in pancreatic β-cells [4]–[6]. Dysregulation of PC expression in liver, adipose tissue or islets is also associated with obesity and type 2 diabetes [7]–[11]. PC deficiency is a rare autosomal recessive phenotype characterized by mild to severe lactic acidemia associated with delayed psychomotor development and death within the first year of life in about one-half the cases [12].

PC is allosterically activated by acetyl-CoA, a signaling molecule that is produced by increased fatty acid oxidation during prolonged starvation. In mammals, the PC gene is transcriptionally regulated by alternate promoters which mediate the production of multiple mRNA isoforms which differ in their 5′-untranslated regions. The PC genes from rat and mouse are well characterized and they are controlled by two promoters namely the proximal and the distal promoters [13]–[15]. The proximal promoter is responsible for production of PC mRNA in the gluconeogenic tissues including liver and kidney, as well as the lipogenic tissues including liver and adipose tissues. The presence of a cAMP-responsive element (CRE) [16]) and a peroxisome proliferator activated receptor response element (PPRE) [17] in the proximal promoter allows liver and adipose tissue, respectively, to produce more PC during prolonged fasting. In contrast, the distal promoter is linked to anaplerosis especially in pancreatic β-cells. The structural region of the human PC gene has been cloned and characterized [18]. However, the regulatory regions of the PC gene that confer tissue-specific expression of PC in humans are not known. Recently, Wang et al [19] reported that unlike the rat and mouse PC genes, the human PC gene is transcribed from three promoters. Herein, we present evidence that similar to the rodent PC genes, the human PC gene is transcribed from two promoters. In addition, we identified some of the important *cis*-acting elements of the distal human PC promoter that direct transcription of PC in beta cells.

## Results and Discussion

### The Human PC Gene is Regulated by Two Promoters and the Distal Promoter is Functional in Pancreatic β-cells

We have previously reported two PC mRNA isoforms with distinct 5′-untranslated regions (UTR) that contain the same coding sequences have been identified in liver and kidney. These two mRNA variants are likely to be generated from alternate transcription from two promoters [13]. In contrast to our study, Wang et al [19] compared the 5′-UTR sequences of three human PC mRNA variants namely, variant 1 (NM_000920.3), 2 (NM_022172.2) and 3 (BC011617.2) deposited at the NCBI database to the genomic sequence of human PC gene and concluded that these variants are alternatively spliced from four 5′-UTR exons, i.e. UE1, UE2, UE3 and UE4, respectively, with the distal, middle and proximal promoters located immediately upstream of exons UE1, UE2 and UE4, respectively [19].

**Figure 1:**
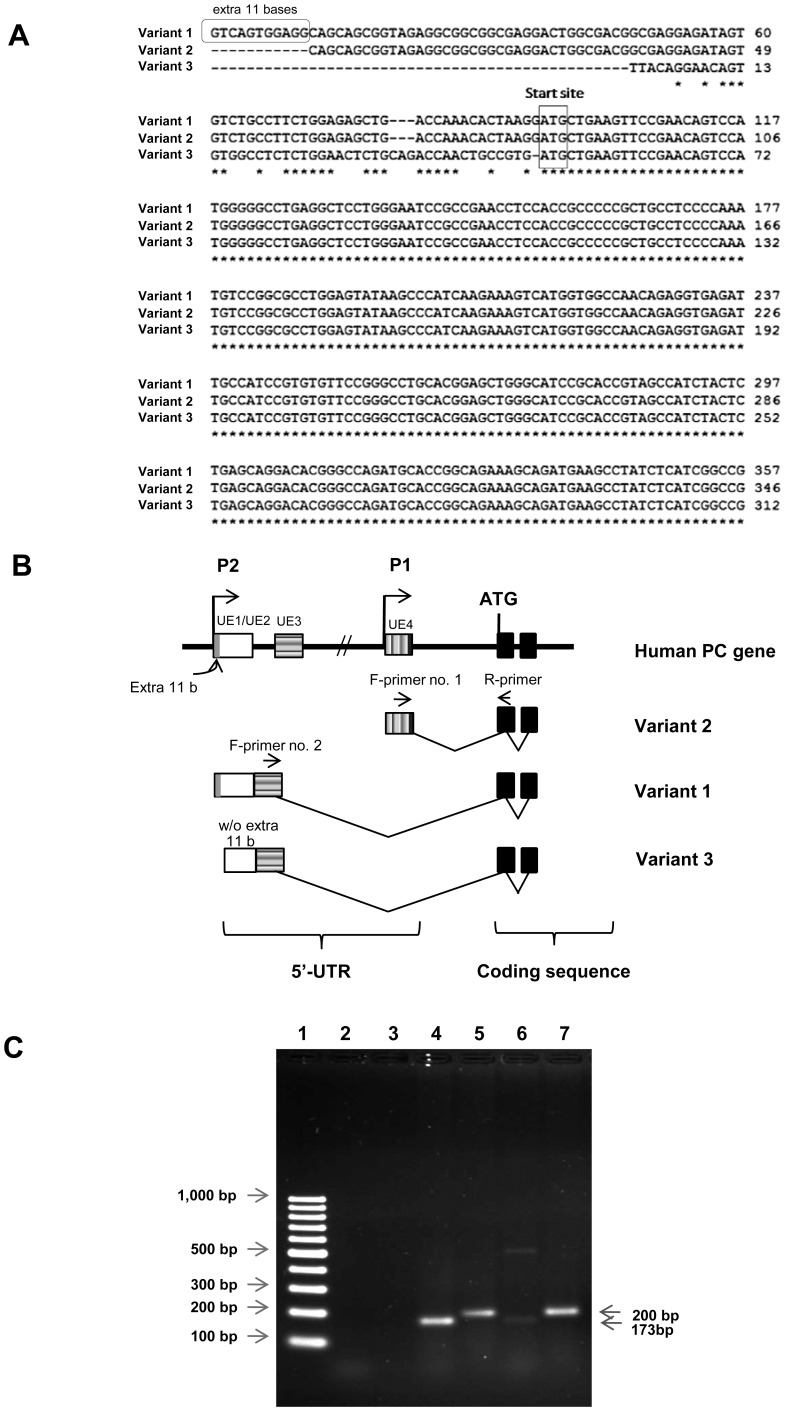
RT-PCR analysis of PC mRNA variants in human liver and human pancreatic islets. (A) Schematic diagram showing alignment of 3 variants of human PC mRNA (GenBank NM_000920.3, NM_022172.2, BC011617.2). (B) Schematic diagram showing the structure of the human PC gene. Two isoforms of human PC mRNA are initiated by two alternative promoters, the proximal (P1) promoter and the distal (P2) promoter. All PC mRNA variants contain the same coding sequences but differ in their 5′-untranslated regions (UTR) produced from different 5′-UTR exons (UE1/UE2, UE3 and UE4) (C) Examination of human PC mRNA in liver and pancreatic islets using RT-PCR. Two sets of primers were used to amplify two different isoforms of human PC mRNA both in human liver and human islets. The 173 bp fragment PCR product of variant 2 and the 200 bp fragment PCR product of variant 1 were amplified by using Primers set no. 1 and primer set no.2, respectively, Lane 1; 1 kb marker, Lane 2; Negative control for primer set no.1, Lane 3; Negative control for primer set no.2, Lane 4; PCR using primer set no.1 and cDNA prepared from human liver, Lane 5; PCR using primer set no.2 and cDNA prepared from human liver, Lane 6; PCR using primer set no.1 and cDNA prepared from human islets, Lane 7; PCR using primer set no.2 and cDNA prepared from human islets.

However, we re-examined the alignment of those three variants and found that variants 1 and 3 share the common 83 nucleotides upstream of the first initiation codon, while variant 1 contains 11 additional nucleotides at its 5′-end (see Figure 1A). Wang et al [19] reported that this extra sequence is derived from an upstream exon, UE1. However, direct comparison of 5′-UTR sequences of variants 1 and 3 with the genomic sequence of the human PC gene clearly showed that these extra 11 nucleotides in variant 1 are located immediately upstream of UE2, thus forming part of this exon. Therefore, it is highly likely that the 11 nucleotide segment in variant 1 could easily be a truncated transcript or result from the use of multiple start sites of the TATA-less genes. In agreement with Wang et al [19], the 5′-UTR sequence of variant 2 is derived from a separate 5′ UTR exon which is located proximal to the first coding exon. The lack of an intron between UE1 and UE2 rules out the possibility that there is a middle promoter located between these two upstream exons as proposed by Wang et al [19]. Based on this new information we revised the structural organization of the human PC gene as follows: the human PC gene contains only three 5′-UTR exons, i.e. UE1/UE2, UE3 and UE4, with the proximal promoter located upstream of UE4 and the distal promoter located upstream of UE1/UE2. Transcription initiated from the proximal promoter produces variant 2 while transcription from the distal promoter produces variants 1 and 3 (Figure 1B). The presence of two alternative promoters of human PC gene appears to recapitulate that of the rat [14] and mouse PC genes [14]. This is in contrast to bovine PC gene which possesses three promoters, the proximal (P1), middle (P2) and distal (P3) promoter [20]. However, there is no report about which of these promoters is highly active in bovine pancreatic β-cells.

Although the two PC mRNA isoforms have been described in liver and kidney [13], [19], it is not known which of these isoform(s) is expressed in human pancreatic islets. To address this question, we performed an RT-PCR analysis of cDNA prepared from human islets using two forward primers that specifically bind to the 5′-UTRs of variant 1 and variant 2 together with a reverse primer that binds to exon 1 (see Figure 1B). With these primers, the amplicons with sizes of 173 bp and 200 bp, representing variant 1 and variant 2 were expected. As shown in Fig. 1C, both primer sets were able to amplify the 173 bp and 200 bp PCR products representing variants 1 and 2 which are produced from both proximal and distal promoters of the human PC gene from HepG2 cDNA (lanes 4 and 5), respectively. This result indicated that both proximal and distal promoters are active in liver. In a sharp contrast, RT-PCR of cDNA prepared from human islets produced a faint band of the 173 bp PCR product amplified by primers set no.1 (lane 6) while primer set no. 2 amplified a strong band of the 200 bp PCR product (lane 7), suggesting that the distal promoter of the human PC gene primarily controls its transcription in human pancreatic islets similarly to rat islets.

### Cloning and Characterization of hP2 Promoter

**Figure 2:**
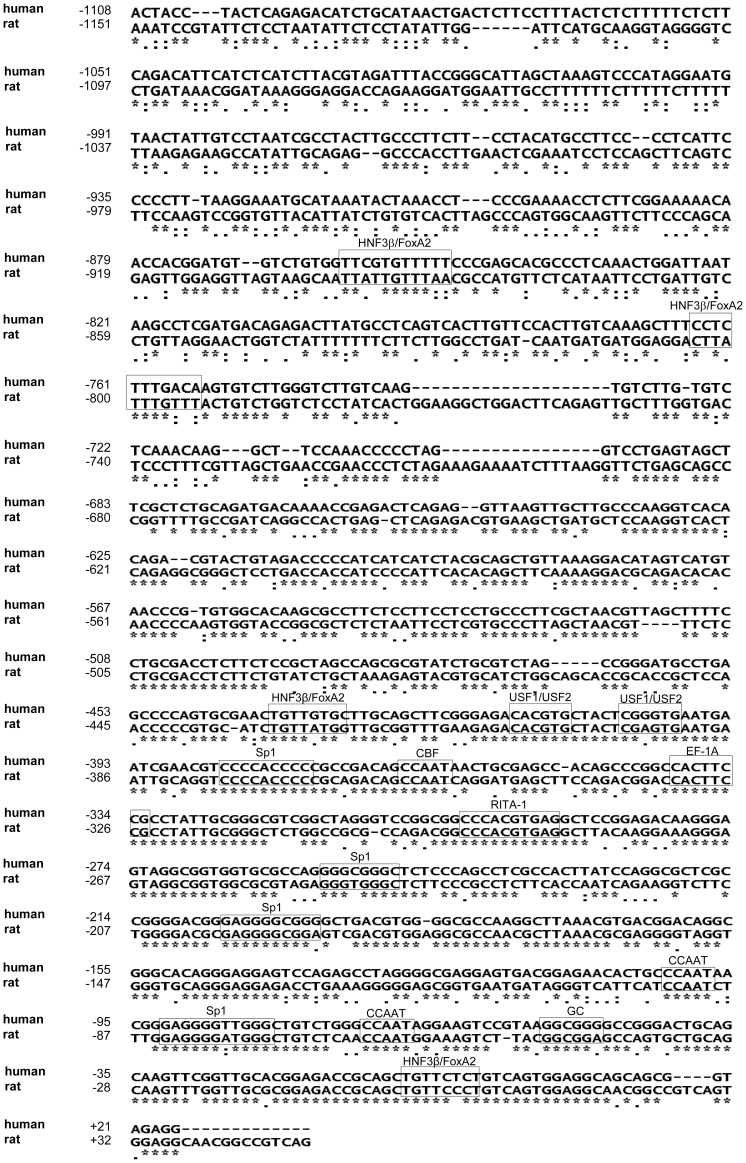
The human PC P2 promoter sequence and its alignment with the rat PC P2 promoter. Boxes represent the putative transcription factor binding sites for Sp1, FoxA2/HNF3β, USF1/2, and CBF. Identical nucleotides between human and rat sequence are symbolized by an asterisk.

To identify the critical *cis*-acting elements that control PC transcription in pancreatic islets, we isolated approximately the 1 kb upstream sequence of exon UE1/2 of the human PC gene which would potentially serve as the distal promoter (hP2) of the PC gene using PCR with the primers designed from the human genome database [21]. A comparison of the nucleotide sequences of the hP2 promoter with the distal promoter of rat PC gene revealed that they are 59.6% similar, with the highest similarity observed within the first 500 nucleotides. The hP2 promoter lacks a canonical TATA box in the first 100 nucleotides but contains two copies of CCAAT boxes and one copy of a GC box located at nucleotide positions −101/−97, −71/−67 and −54/−39, respectively. These features are the characteristic of housekeeping genes [22]. Further analysis of the hP2 promoter sequence using the PROMO database [23] identified several putative transcription factor binding sites including USF1/USF2, Sp1 and HNF3β/FoxA2. These putative binding sites are also conserved in the rat PC gene (Figure 2).

**Figure 3:**
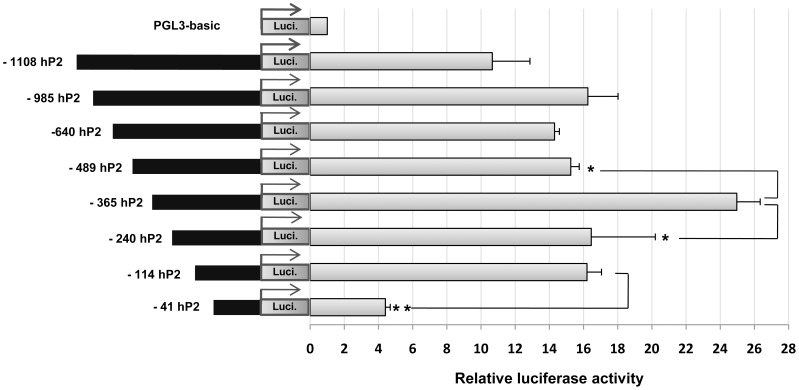
Localization of *cis*-acting elements of the human PC P2 promoter. Transient transfections of 8 constructs containing of the 5′-truncated hP2 promoter into INS-1 832/13 cells were performed to identify the regulatory regions of the hP2 promoter. The basal activity of each 5′-truncated hP2 promoter was calculated from the values of luciferase activity which was normalized with the values of β-galactosidase activity to control for transfection efficiency. The normalized luciferase activity of each P2 construct was compared with the activity of the pGL3-basic vector which was arbitrarily set to 1 and presented as the relative luciferase activity. *P value <0.05, **P value <0.01.

To determine the transcriptional activity of the distal promoter of the human PC gene, a series of 5′-truncated hP2 promoter constructs were generated and used in transient transfection experiments. In this study, eight constructs of the hP2 promoter were transiently transfected into INS-1 832/13 cells. As shown in Fig. 3, deletions of regions −1108 to −985, −640, and −489 did not significantly affect promoter activity. However, when the deletions were made from the region −498 to −365, this resulted in a significant increase of promoter activity, suggesting the presence of a repressor element between these regions. On the other hand, deletions from the region −365 to −240 resulted in a significant decrease in promoter activity, suggesting the presence of (a) positive regulatory element(s) in this region. Further deletion from −240 to −114 did not affect promoter activity. However, deletion to −40 resulted in a dramatic decrease of promoter activity, suggesting the presence of a second positive regulatory element between −114 and −40.

### The −69/−54, −340/−315 Regions of the hP2 Promoter Contain *cis*-acting Elements that Confer Non-beta Cell and Beta-cell Specificity, Respectively

**Figure 4:**
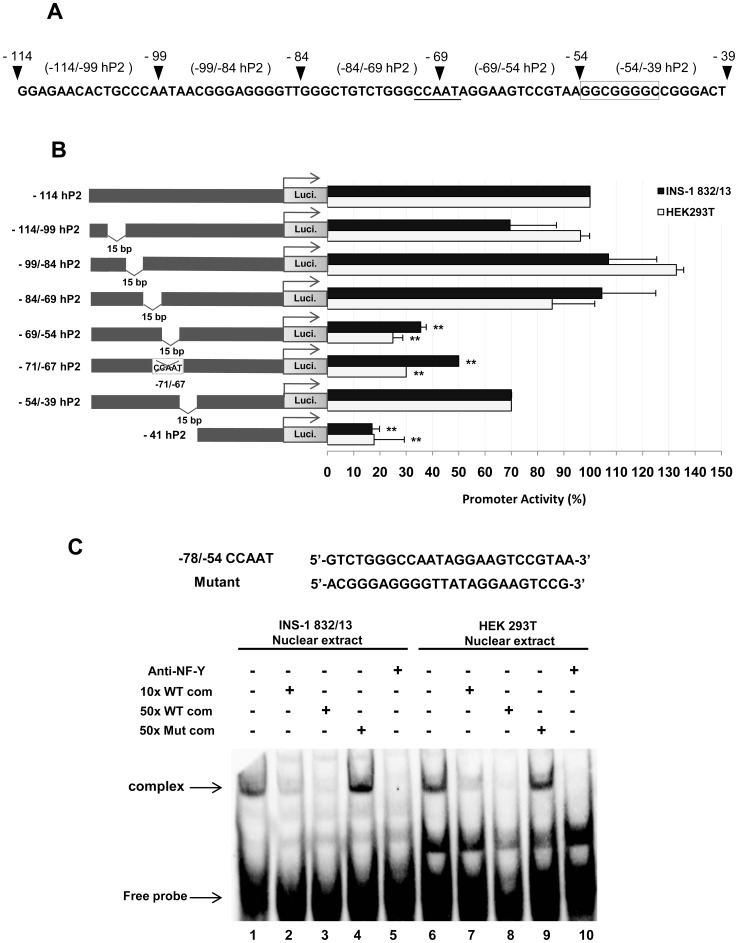
Identification of positive regulatory element(s) located between −114 and −39 of the human PC P2 promoter. (A) Schematic diagram of 15 bp internal deletions of −114/−39 of the human PC P2 promoter. (B) Transient transfections of a series of 15 bp internal deletion constructs into the INS-1 832/13 and non-beta cell HEK293T cell lines were performed to localize the positive regulatory sequence in the human PC P2 promoter. The luciferase activity of each construct was normalized with the β-galactosidase activity. The normalized reporter activity obtained from each construct is shown as a percent relative to those transfected with the wild type −365 hP2 promoter that was arbitrarily set at 100%. *P value <0.05, **P value <0.01. (C) Gel shift and supershift assays of biotin-labeled probe −78 to −54 region of hP2 promoter (−78/−54 CCAAT-probe) using INS-1 832/13 nuclear extract (Lane 1–5) and non-beta cell HEK293T (Lanes 6–10). The nucleotide sequence of wild type and mutant of the hP2 promoter in the −78 to −54 regions are also shown. Lanes 1 and 5 show probes incubated with nuclear extracts from INS-1 832/13 or HEK293T cells; lanes 2 and 6, 10-fold excess wild-type unlabeled oligonucleotides were incubated with nuclear extracts and probes; lanes 3 and 7, 50-fold excess wild-type unlabeled oligonucleotides were incubated with nuclear extracts and probes; lane 4 and 9, 50-fold excess amount of mutant unlabeled oligonucleotides were incubated with nuclear extracts and probes; lanes 5 and 10, nuclear extracts were pre-incubated with anti-NF-Y antibody before the probes were added to the reactions. Arrow represents CCAAT box–NF-Y, complex.

As the first *cis*-acting element which serves as an activator sequence was located between −114 and −41 of the hP2 promoter, a series of 15 bp-internal deletions across this region were generated in order to precisely map the critical element located in this region. These mutant constructs were transiently transfected into both the INS-1 832/13 cell line and the human embryonic kidney cell line, HEK293T. A schematic diagram of 15 bp deletions of the −114/−39 region of the hP2 promoter is shown in Figure 4A. As shown in Figure 4B, transient transfections of −114/−99, −99/−84, −84/−69 deletion mutants did not significantly affect the reporter activity in either cell line. However, deletion of regions between −69 and −54 (−69/−54 hP2) resulted in a dramatic decrease in promoter activity to 35% and 25% of that seen with the INS-1 832/13 and HEK293T cell lines, respectively, suggesting that the −69 to −54 region of the hP2 promoter contains (a) critical *cis*-acting element(s) for basal transcription factors in both the INS-1 832/13 and the HEK293T cell lines. Examination of the nucleotide sequence located between the −69 and-54 of the hP2 construct identified the presence of a CCAAT box located between −71 and −67 (Figure 4B, underlined). To examine whether the dramatic decrease of the luciferase reporter activity observed from the −69/−54 hP2 mutant construct could indeed be attributed to the lack of an intact CCAAT box, we generated another mutant (−71/−67 hP2) in which the whole CCAAT box was deleted. Transient transfection of this mutant construct into INS-1 832/13 and HEK293T cells resulted in a marked reduction of promoter activity in both cell lines, similar to that of the −69/−67 hP2 mutant construct, suggesting that the −71/−67 CCAAT box is crucial for maintaining basal activity of the P2 promoter both in INS-1 832/13 and HEK293T cells. Deletion of the regions between −54 to −39 (−54/−39 hP2 construct), resulted in a marginal reduction of the reporter activity in both cell lines. Examination of the nucleotide sequence surrounding this region identified the presence of a GC-box, which is also found in the identical position in the distal promoter of the rat PC gene. This GC-rich region serves as a binding site for ubiquitous transcription factors Sp1/Sp3 [24]. Mutation of this similarly located GC-box in the rat gene resulted in a reduction of the reporter gene activity to a greater extent (80% reduction) than mutation of this sequence in the human gene [24], suggesting the rat and human PC genes are regulated differently via the GC-box.

A CCAAT box serves as a potential binding site for the nuclear factor Y (NF-Y) [25] and binding of this factor to this sequence is essential for transcriptional activation of TATA-less genes [26], [27]. We confirmed this by performing gel shift experiments. As shown in Figure 4C, incubation of the –78/−54 probe harboring the −71/−67 CCAAT box with a nuclear extract of INS-1 832/13 cells produced a predominant DNA-protein complex (lane 1). This complex was readily competed off with 10x and 50x unlabelled WT double-stranded oligonucleotide (lanes 2–3), but was not competed off with an unrelated double stranded oligonucleotide sequence (lane 4). Incubation of anti-NF-Y polyclonal antibody prevented the formation of a DNA-protein binding complex (lane 5). A similar result was obtained when a nuclear extract of HEK293T cells was used in the experiment (lanes 6–10). These data indicate that NF-Y is a transcription factor that directs PC transcription via the −71/−67 CCAAT box in both cell lines. Although this CCAAT box appears to be conserved in the distal promoter of both the rat and human PC genes, it serves different roles in transcriptional regulation in the two genes. In the distal promoter of rat PC gene, this CCAAT box serves a repressor element, while in the human PC gene, this sequence clearly acts as an activator sequence. This dual function of NF-Y being both transcriptional activator and repressor is not totally unexpected as this depends on the promoter context [28]. NF-Y can possess a repressor activity if its recognition sequence is overlapped with the nearby activator binding sequence, antagonizing activator function [29]–[31]. The above data indicate that although the *cis*-acting elements including the CCAAT box and the GC-box are found in similar locations for both human and rat PC genes, their actions are substantially different. In the rat PC gene, the CCAAT box serves as a repressor element that somewhat antagonizes the GC-box function [24], while in the human PC gene, the CCAAT box sequence clearly acts as an activator element. As the GC-box in the human PC gene is not as strong an activator as in the rat PC gene, it appears that in the human PC gene, the upstream CCAAT box acts as an activator sequence to maximize transcription.

**Figure 5:**
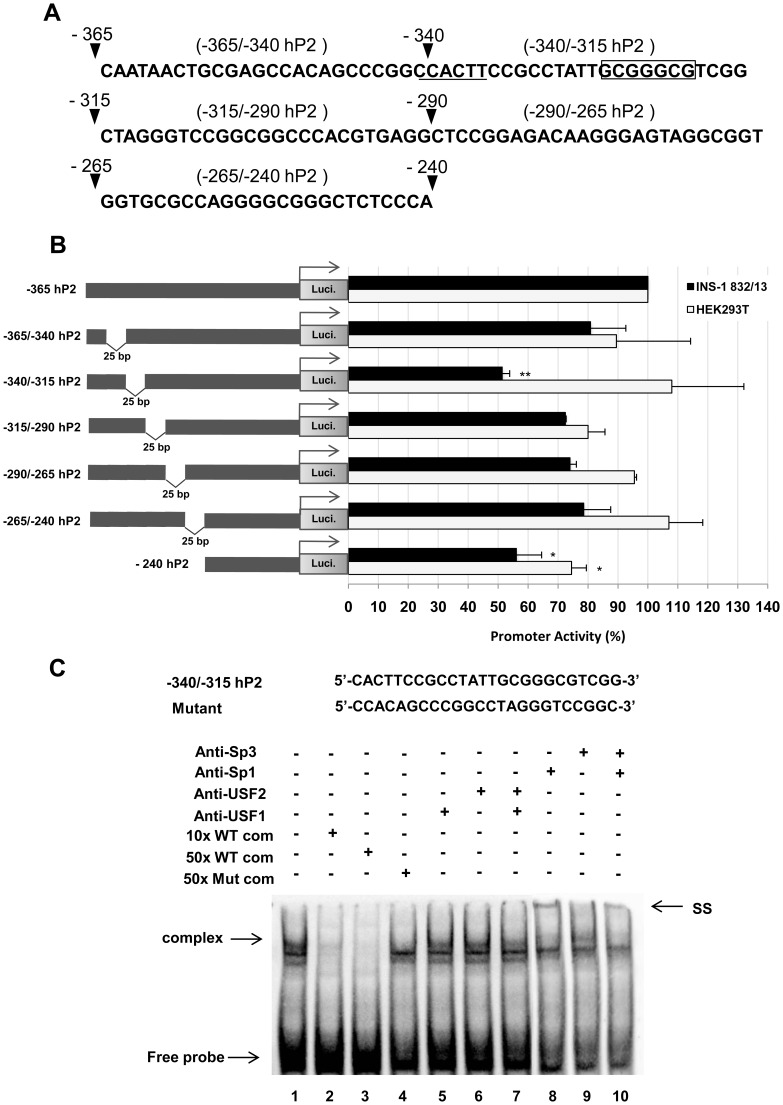
Identification of positive regulatory element(s) located between −365 and −240 of the human PC P2 promoter. (A) Schematic diagram of 15 bp internal deletions of −114/−39 the human PC P2 promoter. (B) Transient transfections of a series of 25 bp internal deletion constructs into the INS-1 832/13 cell line and non-beta cell HEK293T cell line were performed to identify the positive regulatory sequences in the hP2 promoter. The luciferase activity of each construct was normalized with β-galactosidase activity. The normalized reporter activity obtained from each construct is shown as a percent relative to those transfected with the wild type −365 hP2 promoter, which was arbitrarily set at 100%. *P value <0.05, **P value <0.01. (C) Gel shift and supershift assays of the biotin-labeled probe of the −78 to −54 region of the hP2 promoter (−340/−315 hP2 probe) using an INS-1 832/13 nuclear extract. The nucleotide sequences of the wild type and mutant of the hP2 promoter −78 to −54 regions are also shown. Lane 1 probes incubated with nuclear extracts from INS-1 832/13; lanes 2–3, 10-fold or 50-fold excess wild-type unlabeled oligonucleotides were incubated with nuclear extracts and probes; lane 4, 50-fold excess amount of mutant unlabeled oligonucleotides were incubated with nuclear extracts and probes; lanes 5–7, nuclear extracts were pre-incubated with anti-USF1 or anti-USF2 or both, respectively, before the probes were added to the reactions. Lanes 8–10, nuclear extracts were pre-incubated with anti-Sp1 or anti-Sp3 or both, respectively, before the probes were added to the reactions. Arrow represents DNA-protein complex, SS = supershift band.

**Figure 6:**
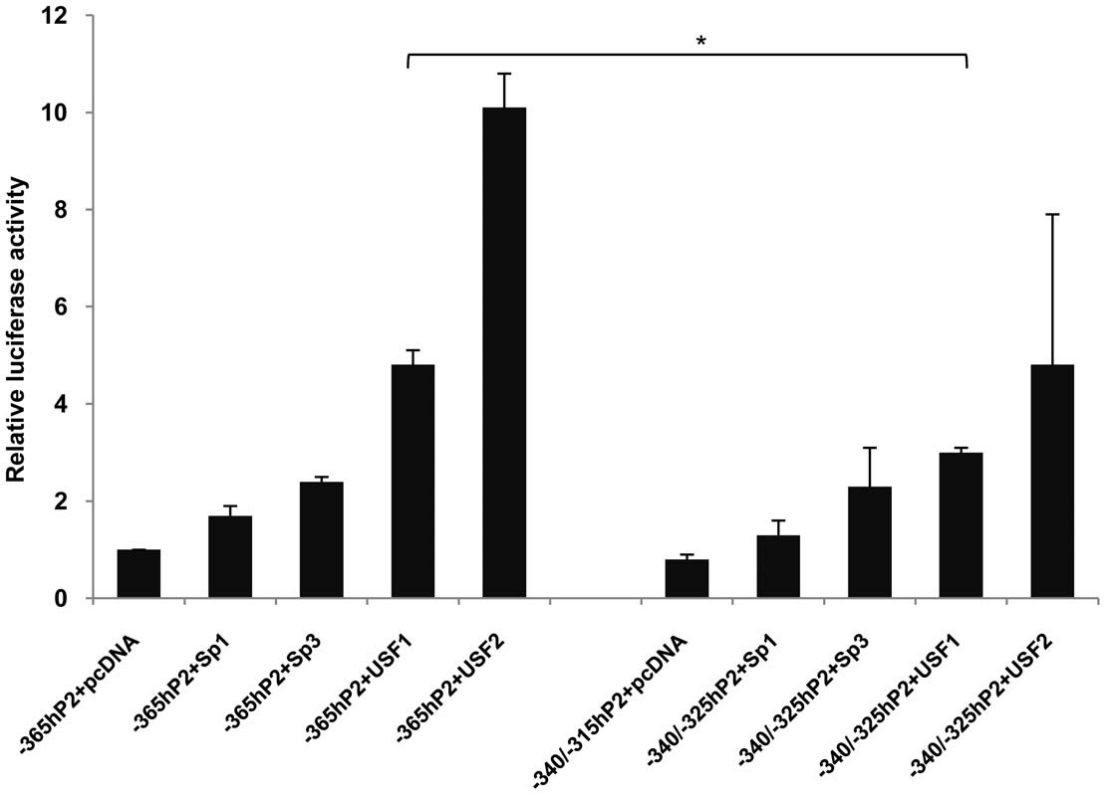
Transactivation of a WT −365 human PC P2 luciferase reporter construct and its mutant by Sp1, Sp3, USF1 or USF2. WT −365 hP2 or −340/−315 hP2 constructs were co-transfected with an empty vector (pcDNA3) or a plasmid overexpressing Sp1, Sp3, USF1 or USF2 into the INS-1 832/13 cell line, and the luciferase activities measured. The luciferase activity was normalized to β-galactosidase activity and expressed as relative luciferase activity. Relative luciferase values obtained from co-transfecting cells with wild type (−365 hP2) or its mutant (−340/−315 hP2) and plasmid overexpressing Sp1, Sp3, USF1 or USF2 were presented as fold change relative to those obtained from those co-transfected with WT with empty vector (pcDNA3) which was arbitrarily set at 1. *p≤0.01.

To more precisely localize the positive regulatory sequences between −365 and −240 of hP2, 25-bp internal deletions of the −365/−240 hP2 promoter were made. Five mutants harboring 25 bp internal deletions across the −365 to −240 regions (−365/−340, −340/−315, −315/−290, −290/−265 and −265/−240 hP2) were generated and transfected into both INS-1 832/13 and HEK293T cells. A schematic diagram of the 25 bp deletions of the −365/−240 hP2 promoter region is shown in Figure 5A. As shown in Figure 5B, transient transfection of −340/−315 hP2 mutant construct markedly reduced the reporter gene activity to 50% of the −365 hP2 promoter in INS-1 832/13 cells, while no reduction of reporter gene activity was observed in HEK293T cells. In contrast, deletion of other regions did not affect the promoter activity when compared to the wild type −365 hP2 promoter in either cell line. These data suggest the presence of a tissue specific *cis*-acting element(s) located between −340 and −315 in the hP2 promoter. To identify which transcription factors might bind to this element we performed gel shifts experiments in which double stranded oligonucleotides harboring −340/−315 were incubated with a nuclear extract of INS-1 832/13 cells. As shown in Figure 5C, a strong DNA-protein complex was observed. Examination of nucleotide sequences between −340 and −315 identifies an E-box, a binding site for USF [32], located between –341 and −336 and a GC-box and a binding site for Sp1/Sp3, located between –326 and −320, respectively. Incubation of an anti-Sp1 antibody in the binding reaction produced a weak super-shift band, while incubation in the presence of anti-Sp3, anti-USF1 or anti-USF2 antibodies had no effect on the DNA-protein complex formation, indicating that these three factors may not attribute to the binding to this sequence. To confirm the gel shift experiment, we performed a transactivation assay in which the wild type (−365 hP2) construct was co-transfected with plasmid overexpressing Sp1, Sp3, USF1 or USF2, and the luciferase activities were measured. As shown in Figure 6, co-transfection of Sp1 or Sp3 resulted in only 1.5-fold or 2-fold increase in the reporter gene activity, consistent with a poor or lack of evidence of their binding to the −340/−315 sequence shown in Figure 5C. Mutation of this sequence also had no effect on the expression of the reporter gene. The poor remaining Sp1 and Sp3-mediated transcriptional activation of the human PC promoter may be attributed to the GC box located at −54/−39 (Figure 4A). Despite the lack of evidence of binding of USF1 or USF2 to the E-box located between −340/−315, overexpression of USF1 or USF2 resulted in approximately 5-fold or 10-fold increase in the promoter activity. However, deletion of the sequences located between −340 and −315 did not significantly affect USF1- or USF2- mediated transcriptional activation of the human PC promoter, suggesting that the transactivation by these two factors may be mediated through the downstream E-boxes.

In summary we have shown that: (i) the human PC gene possesses only two promoters, P1 and P2, which mediate transcription of the human PC gene similar to the rat and mouse genes; (ii) the P1 and P2 promoters are active in hepatocytes while only the P2 promoter is active in pancreatic β-cells; (iii) both CCAAT box and GC-boxes serve as activator sequences in β-cells; (iv) a *cis*-acting element located between −340/−315 serves as binding site for β-cell specific transcription factor.

## Materials and Methods

### Reverse Transcriptase-polymerase Chain Reaction (RT-PCR)

To identify the predominant isoform of the human PC mRNA in pancreatic beta cells, RT-PCR using human cDNA prepared from human islets and liver was performed. In this experiment, two sets of primers directed to various 5′-UTR exons of the PC gene (GenBank NM_000920.3, NM_022172.2, BC011617.2) were designed and used in RT-PCR. Both primer sets consisted of the same sequence of the reverse primer (R-primer) and a different sequence of the forward primer (F-primer). The F-primer set no. 1 (5′-ACCAACTGCCGTGATGCTGA-3′) was designed to bind to the 5′-UTR of variant 2 of human PC mRNA which is transcribed by the proximal promoter while the F-primer set no. 2 (5′-GATAGTGTCTGCCTTCTGGAGAGC-3′) was designed to bind to the 5′-UTR region of variant 3 of the human PC mRNA which is transcribed by the distal promoter. The R-primer (5′-ACACACGGATGGCAATCTCACC-3′) was designed to bind to exon 1 of human PC mRNA [33]. Tissues were homogenized with a Qiashredder (Qiagen) (islets) or using a Potter–Elvehjem homogenizer (liver) and RNA was prepared using the RNeasy Mini kit (Qiagen). On-column DNase digestion was performed using the Qiagen RNase-Free DNase Set. cDNA was made with randomized primers with the Retroscript kit (AM1710) (Applied Biosystems). Quantitative PCR was performed on a BioRad MyIQ Real Time Detection System with SYBR Premix Ex Taq (RR041Q) (Takara). Human liver RNA was from a 51-year old male (Clontech, catalog number 636531) and a liver surgical specimen from a person (of unknown age and gender due to privacy protection) [34]. The PCR was carried out in a 20 µl-reaction mixture containing 2 µl of cDNA, 1x PCR reaction buffer (20 mM Tris-HCl pH 8.4, 50 mM KCl), 0.2 µM of each primer, 100 µM of each dNTP, 2 mM MgCl_2_, and 1 unit *Taq* DNA polymerase. The PCR profile consisted of an initial denaturation at 94°C for 5 min followed by 35 cycles of denaturation at 95°C for 30 sec, annealing at 55°C for 30 sec, and extension at 72°C for 45 sec, and final extension at 72°C for 10 min.

### Cloning of hP2 Promoter Linked Luciferase Gene Constructs

The 1,108 bp fragment of the hP2 promoter was cloned from genomic DNA isolated from HepG2 cells using the hP2-forward primer (5′-GGTACCACTACCTACTCAGAGACATCTGC-3′; underline indicates a *Kpn*I restriction site) and the hP2-reverse primer (5′-CTCGAGGTCCTCGCCGCCGCCTCTACC-3′; underline indicates a *Xho*I restriction site). The PCR product was then ligated to the pGEM-T Easy vector (Promega) and sequenced. The clone with the correct sequence of the hP2 promoter was excised from the pGEM-T easy vector with *Kpn*I and *Xho*I sites and ligated to the equivalent sites of the pGL3-basic vector (Promega) to generate a hP2-luciferase reporter construct. 5′-truncated hP2 promoter constructs comprising 985, 640, 365, 240, 114, and 41 nucleotides of the hP2 promoter were generated by PCR using a full length hP2 promoter-luciferase construct as a template. The forward primers containing a *Kpn*I site at their 5′-ends and the reverse primer containing an *Xho*I site at the 3′-end were designed. The PCR products were then ligated into the pGEM-T Easy vector and sequenced. The correct sequences of 5′-truncated hP2 promoter were excised with *Kpn*I and *Xho*I and ligated to the equivalent sites of the pGL3-basic vector. Primers used for cloning of 5′-truncated hP2 promoters are shown in Table 1. For the construction of a 489 bp fragment of hP2 promoter, the promoter was generated by double digestion of the full length hP2 promoter-luciferase construct with *Nhe*I and *Xho*I. The 489 bp fragment of the hP2 promoter was then re-ligated into the *Nhe*I and *Xho*I site of the pGL3-basic vector.

**Table 1:** Oligonucleotides used for construction of 5′-trucated hP2 promoter.

Primer name	Sequences (5′ to 3′)	Length (bp)
−985 bp hP2-F	GGTACC TTGTCCTAATCGCCTACTTGC	27
−640 bp hP2-F	GGTACC TTGCCCAAGGTCACACAGACG	27
−365 bp hP2-F	GGTACC CAATAACTGCGAGCCACAGC	26
−240 bp hP2-F	GGTACC GCCTCGCCACTTATCCAGGCG	27
−114 bp hP2-F	GGTACC GGAGAACACTGCCCAATAACG	27
−41 bp hP2-F	GGTACC CTGCAGCAAGTTCGGTTGCACG	28
−39 bp hP2-R	CTCGAG GTCCTCGCCGCCGCCTCTACC	27

* Restriction enzyme recognition sites are underlined.

### Site-directed Mutagenesis

Site-directed mutagenesis using the QuikChange site-directed mutagenesis kit (Agilent Technologies) was performed to generate 5, 15 and 25 nucleotide internal deletion mutants of the hP2 promoter constructs. The mutagenesis reaction was carried on in a total volume of a 50 µl-reaction mixture containing 300 ng of DNA template, 125 ng of each mutagenic oligonucleotide primer, 10 mM KCl, 10 mM (NH_4_)_2_SO_4_, 20 mM Tris-HCl pH 8.8, 2 mM MgSO_4_, 0.1% TritonX-100 and 0.1 mg/ml nuclease-free bovine serum albumin (BSA), 200 µM dNTP mix, and 2.5 U of *Pfu*Turbo polymerase (Stratagene-Agilent Technologies). The amplification profile consisted of an initial denaturation at 95°C for 30 sec followed by 20 cycles of denaturation at 95°C for 30 sec, annealing at 55°C for 1 min, and extension at 68°C for 10 min. The primers used for site-directed mutagenesis are shown in Tables 1 and 2. The correct mutant constructs were verified by automated nucleotide sequencing. The corrected clones with 5, 15 or 25 nucleotide deletion were double digested with *Kpn*I and *Xho*I and re-ligated into the pGL3 basic vector digested with the same enzymes.

**Table 2:** Oligonucleotides used for generation of 25 bp deletion of −365/−240 hP2, 15 bp deletion of −114/−39 hP2 and 5 bp deletion of −114/−39 hP2 promoter constructs.

Primer name	Sequences (5′ to 3′)	Construct name
−365/−340 hP2-F	TCGATTGGTACCCACTTCCGCCTA	−365/−340 hP2
−365/−340 hP2-R	TAGGCGGAAGTGGGTACCAATCGA	
−340/−315 hP2-F	CCACAGCCCGGCCTAGGGTCCGGC	−340/−315 hP2
−340/−315 hP2-R	GCCGGACCCTAGGCCGGGCTGTGG	
−315/−290 hP2-F	TGCGGGCGTCGGCTCCGGAGACAA	−315/−290 hP2
−315/−290 hP2-R	TTGTCTCCGGAGCCGACGCCCGCA	
−290/−265 hP2-F	GCCCACGTGAGGGGTGCGCCAGGG	−290/−265 hP2
−290/−265 hP2-R	CCCTGGCGCACCCCTCACGTGGGC	
−265/−240 hP2-F	GGAGTAGGCGGTGCCTCGCCACTT	−265/−240 hP2
−265/−240 hP2-R	AAGTGGCGAGGCACCGCCTACTCC	
−114/−99 hP2-F	TCGATAGGTACCATAACGGGAGGG	−114/−99 hP2
−114/−99 hP2-R	CCCTCCCGTTATGGTACCTATCGA	
−99/−84 hP2-F	GAACACTGCCCAGGGCTGTCTGGG	−99/−84 hP2
−99/−84 hP2-R	CCCAGACAGCCCTGGGCAGTGTTC	
−84/−69 hP2-F	ACGGGAGGGGTTATAGGAAGTCCG	−84/−69 hP2
−84/−69 hP2-R	CGGACTTCCTATAACCCCTCCCGT	
−69/−54 hP2-F	CTGTCTGGGCCAGGCGGGGCCGGG	−69/−54 hP2
−69/−54 hP2-R	CCCGGCCCCGCCTGGCCCAGACAG	
−71/−67 hP2-F	GGGCTGTCTGGGAGGAAGTCCGTA	−71/−67 hP2
−71/−67 hP2-R	TACGGACTTCCTCCCAGACAGCCC	
−54/−39 hP2-F	GGAAGTCCGTAAGCAGCAAGTTCG	−54/−39 hP2
−54/−39 hP2-R	CGAACTTGCTGCTTACGGACTTCC	

### Cell Culture and Transfection

INS-1 832/13 cells [35] were maintained in RPMI 1640 supplemented with 28 mmol/l NaHCO_3_, 1 mM sodium pyruvate (Gibco), 50 µM β-mercaptoethanol, 10% (v/v) heat-inactivated fetal bovine serum (Gibco), and 50 units/l penicillin/streptomycin at 37°C in 5% CO_2_. In the transfection experiments, 2×10^5^ cells were seeded in 24-well plates and were cultured in 0.5 ml of antibiotic-free DMEM (Dulbecco’s modified Eagle’s medium; Gibco) containing 10% fetal bovine serum for 24 h before transfection. Cells were transfected with 250 ng of the luciferase reporter constructs and 250 ng of pRSV-β-gal plasmid expressing β-galactosidase using Lipofectamine™ 2000 reagent (Invitrogen). For transactivation assays, 250 ng of plasmids overexpressing Sp1, Sp3 [36], USF1 or USF2 [24] were also included with the luciferase reporter construct and pRSV-β-gal plasmid. The transfected cells were maintained in the antibiotic-free DMEM at 37°C for 48 h. For the transfection of the non-beta cell line, the human embryonic kidney cell line (HEK293T) was grown in DMEM supplemented with 10% heat-inactivated fetal bovine serum, and 50 units/L penicillin/streptomycin at 37°C in 5% CO_2_. The transfections were carried out as described for the INS-1 832/13 cells [37], except that the cells were seeded in 24-well plates at a density of 4×10^5^ cells. The luciferase reporter assays were performed using the luciferase reporter assay system (Promega), while the β-galactosidase assay was performed using ONPG as substrate.

### Electrophoretic Mobility Shift Assay (EMSA)

1×10^7^ of INS-1 832/13 cells were harvested for preparation of nuclear extracts. The cells were washed with PBS and resuspended in 1 ml of nuclear extraction buffer I (10 mM HEPES pH7.9, 1.5 mM MgCl_2_, 10 mM KCl, 0.5 mM DTT and 1x protease inhibitor cocktail (Roche) at 4°C for 1 min. The nuclei were centrifuged at 3,000 g at 4°C for 1 min before resuspended in 100 µl nuclear buffer 2 (20 mM HEPES, pH7.9, 25% (v/v) glycerol, 420 mM NaCl, 1.5 mM MgCl_2_, 0.2 mM EDTA and 0.2 mM PMSF) and incubated on ice for 5 min. The nuclear lysate was centrifuged at 3,000 g for 5 min at 4°C and the supernatant was kept at −80°C and used for EMSA.

The 5′-end labeled biotinylated oligonucleotide was synthesized by BioBasic (Canada) and annealed with the unlabelled complementary strand oligonucleotide. The oligonucleotides used in EMSA are listed in Table 3. The DNA-protein binding assay was carried out in a 20 µl-reaction mixture containing 1x binding buffer (25 mM HEPES, pH7.9), 25% (v/v) glycerol, 420 mM NaCl, 1.5 mM MgCl_2_, 10 mM KCl, 0.2 mM EDTA, 0.5 mM DTT and 0.2 mM PMSF, 10 µg of nuclear extract, 2 µg of poly dI-dC and 120 fmole of biotinylated double stranded oligonucleotide at 4°C for 30 min. For supershift assays, 1 µg of anti-Sp1 (sc-59), anti-Sp3 (sc-644), anti-USF1 (sc-22) or anti-USF2 (sc-862) polyclonal antibody (SantaCruz Biotech) was included in the binding reaction. The DNA-protein complexes were analyzed by 5% non-denaturing polyacrylamide gel electrophoresis followed by electroblotting. The bands of DNA-protein interaction were detected using LightShift Chemiluminescent EMSA kit (Pierce). The image was captured using Gel Doc System (GeneTools).

**Table 3:** Oligonucleotides used in EMSA.

Oligonucleotide	Sequence (5′- 3′)	Probe/competitor
−78/−54 CCAAT-F*	GTCTGGGCCAATAGGAAGTCCGTAA	Probe/WT competitor
−78/−54 CCAAT-R	TTACGGACTTCCTATTGGCCCAGAC	
−78/−54 CCAAT-MuF	ACGGGAGGGGTTATAGGAAGTCCG	Mutant competitor
−78/−54 CCAAT-MuR	CGGACTTCCTATAACCCCTCCGT	
−340/−315 hP2-F*	CACTTCCGCCTATTGCGGGCGTCGG	Probe/WT competitor
−340/−315 hP2-R	CCGACGCCCGCAATAGGCGGAAGTG	
−340/−315 hP2-MuF	CCACAGCCCGGCCTAGGGTCCGGC	Mutant competitor
−340/−315 hP2-MuR	GCCGGACCCTAGGCCGGGCTGG	

* 3′ labeled with biotin.
